# Detection of a novel pathogenic variant in *KCNH2* associated with long QT syndrome 2 using whole exome sequencing

**DOI:** 10.1186/s12920-024-01900-z

**Published:** 2024-05-07

**Authors:** Erfan Kohansal, Niloofar Naderi, Amir Farjam Fazelifar, Majid Maleki, Samira Kalayinia

**Affiliations:** 1 Rajaie Cardiovascular Medical and Research Center, Iran University of Medical Sciences, Tehran, Iran; 2 Cardiogenetic Research Center, Rajaie Cardiovascular Medical and Research Center, Iran University of Medical Sciences, Tehran, Iran

**Keywords:** Long QT syndrome, KCNH2, Whole exome sequencing, Mutation

## Abstract

**Background:**

Long QT syndrome (LQTS) is a cardiac channelopathy characterized by impaired myocardial repolarization that predisposes to life-threatening arrhythmias. This study aimed to elucidate the genetic basis of LQTS in an affected Iranian family using whole exome sequencing (WES).

**Methods:**

A 37-year-old woman with a personal and family history of sudden cardiac arrest and LQTS was referred for genetic study after losing her teenage daughter due to sudden cardiac death (SCD). WES was performed and variants were filtered and prioritized based on quality, allele frequency, pathogenicity predictions, and conservation scores. Sanger sequencing confirmed segregation in the family.

**Results:**

WES identified a novel heterozygous frameshift variant (NM_000238.4:c.3257_3258insG; pGly1087Trpfs*32) in the *KCNH2* encoding the α-subunit of the rapid delayed rectifier potassium channel responsible for cardiac repolarization. This variant, predicted to cause a truncated protein, is located in the C-terminal region of the channel and was classified as likely pathogenic based on ACMG guidelines. The variant was absent in population databases and unaffected family members.

**Conclusion:**

This study reports a novel *KCNH2* frameshift variant in an Iranian family with LQTS, expanding the spectrum of disease-causing variants in this gene. Our findings highlight the importance of the C-terminal region in KCNH2 for proper channel function and the utility of WES in identifying rare variants in genetically heterogeneous disorders like LQTS. Functional characterization of this variant is warranted to fully elucidate its pathogenic mechanisms and inform personalized management strategies.

## Introduction

Long QT syndrome (LQTS) is a cardiac disorder characterized by abnormalities in myocardial repolarization, which can lead to life-threatening arrhythmias such as torsades de pointes (TdP) [1]. Congenital LQTS is primarily caused by mutations in genes encoding cardiac ion channels, leading to impaired repolarization of the myocardium [2]. The prevalence of congenital LQTS varies depending on the population studied, with estimates ranging from 1 in 2,500 to 1 in 7,000 individuals [3]. Several genetic variants have been identified in LQTS with *KCNQ1*, *KCNH2*, and *SCN5A* being the most common genes involved [2]. The genotype of the LQTS influences the clinical course, with a higher risk of cardiac events observed in individuals with mutations at the LQT1 or LQT2 locus compared to those with mutations at the LQT3 locus [4].

LQT2 accounts for approximately 35–45% of cases [5, 6] and if not treated, can manifest clinically as TdP, ventricular fibrillation (VF), or sudden cardiac death (SCD) [7]. LQT2 is mainly caused by loss-of-function mutations in the alpha subunit of the voltage-dependent potassium channel known as hERG or Kv11.1 (coded by *KCNH2* gene), which underlies the rapidly activating delayed rectifier K^+^ current (I_Kr_) in the heart [8]. These loss-of-function variants lead to reduced IKr, prolonging the cardiac action potential duration and increasing the risk of ventricular arrhythmias.

The C-terminal region of the hERG potassium channel, particularly the cyclic nucleotide-binding domain (CNBD), has been shown to play a crucial role in regulating the channel’s gating properties. There is compelling evidence for a direct interaction between the N-terminal PAS domain and the CNBD, suggesting that this interaction is essential for the slow deactivation gating characteristic of hERG channels [9]. Disruption of this interaction, either through deletions or mutations in the C-terminal region, can lead to faster deactivation rates [9]. Considering the critical role of hERG channels in repolarization of the cardiac action potential, alterations in their deactivation kinetics due to C-terminal mutations may contribute to the pathogenesis of LQTS [10]. Understanding the functional consequences of novel variants in the C-terminal region of KCNH2 may provide valuable insights into the genotype-phenotype correlations in LQT2 patients and inform the development of targeted therapeutic strategies.

In this study, we aimed to use whole-exome sequencing (WES) to identify the genetic cause of LQT syndrome in an Iranian family with suspected LQT2. By focusing on the C-terminal region of KCNH2, we sought to expand the spectrum of disease-causing variants and contribute to the growing body of evidence supporting the critical role of this region in the pathogenesis of LQT2.

## Materials and methods

### Clinical investigation

**Fig. 1:**
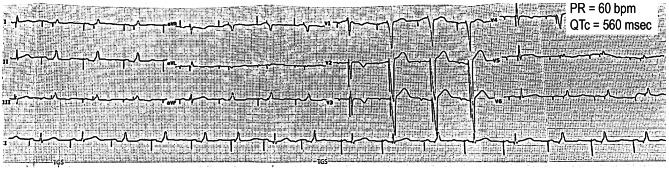
The Proband’s electrocardiogram (ECG). The 12-lead ECG demonstrates the proband has corrected QT (QTc) interval of 560 milliseconds indicative of LQTS diagnosis

A 37-year-old woman (proband) diagnosed with LQTS and sick sinus syndrome was referred to the Rajaie Cardiovascular, Medical and Research Center, Tehran, Iran in 2023 for genetic counseling and further evaluation. The proband had previously lost a 14-year-old daughter due to SCD. The proband and her deceased daughter, both had normal laboratory data and echocardiography findings, but prolonged QT intervals on electrocardiography (Fig. 1). Holter Electrocardiogram of the proband also recorded no significant event. Blood samples were collected from the proband and her spouse. No previously collected sample from the deceased daughter was available, as she had passed away prior to the initiation of this study.

#### Informed consent

was obtained from the proband and her spouse, and the study was conducted in accordance with the Declaration of Helsinki. The study protocol was reviewed and approved by the Research Ethics Committee of the Rajaie Cardiovascular, Medical and Research Center, Iran University of Medical Sciences, Tehran, Iran (IR.RHC.REC.1402.003).

### Exome sequencing

Genomic DNA was extracted from family members whole blood using the standard salting out procedure. Purified DNA concentration and quality were determined by Nanodrop2000 (Thermo Scientific). WES was performed on proband genomic DNA (10 ng) using the HiSeq6000 sequencer (Illumina, San Diego, CA, USA) at Macrogen (Amsterdam, Netherland). The raw sequencing data (fastq file) was processed by Rajaie Cardiovascular Medical and Research Center, Tehran, Iran. Fastq sequence file was checked with FastQC software (v.0.12.0) and aligned and mapped against the human reference sequence (GRCH37/hg19) with the Burrows-Wheeler Aligner (BWA) (v.0.6). Variant calling was performed by using the SAMtools software (v.0.1.x). Annotation of variants were occurred by using ANNOVAR software. In conclusion filtering of variants was done according to their quality and minor allele frequency (MAF < 0.05). Prioritization of variants was prepared according variant type, conservation (GERP) and the scores from prediction tools (CADD, PolyPhen2 and SIFT).

### Mutation validation

For variant validation and family segregation analysis, Sanger sequencing was performed. Amplification corresponding to exon 14 was carried out on a SimpliAmp™ thermal cycler (Applied Biosystems™, Massachussets, USA) using 5′-CTGATGGAGGACTGCGAG-3′ (Forward Sequence) and 5′-GGCTCTTCAGGCGATGCT-3′ (Reverse Sequence). Finally, the Sanger sequencing was performed on an ABI3500 DNA sequencer 96 capillary type (Thermo Fisher Scientific, Waltham, MA, USA). The chromatograms were analyzed using the BioEdit software.

## Results

### Genetic findings

**Fig. 2:**
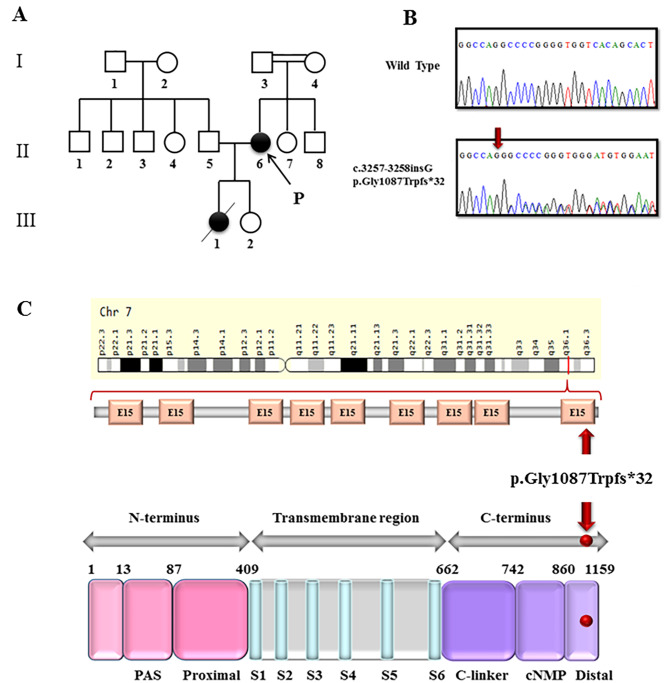
(**A**) The LQTS Family pedigrees from an Iranian family. Arrow (P) denotes proband. Filled symbols indicate clinically and genetically affected individuals. Her daughter died suddenly at 16 years old (cross). (**B**) DNA sequencing analysis: Sanger sequence analysis of the PCR-amplified genomic DNA showing heterozygous introduction of NM_000238.4:c. Gly1087Trpfs*32. These results show that the variant in *KCNH2* gene is inherited from mother (proband) to her daughter. (**C**) Up: a schematic view of the *KCNH2* gene. The image depicts the location of *KCNH2* on the chromosome and the variant location in the exon. The orange boxes are exons. Down: schematic Representation of Voltage-Gated Potassium Channel (Secondary structure of the *KCNH2* protein) and the location of frameshift mutation (p. Gly1087Trpfs*32): The a-subunit of the HERG cardiac potassium Channel is consisted of a cytoplasmic N-terminus, 6 α-helical transmembrane segments are labeled S1–S6, interdomain links, extracellular P-loops (between S5 and S6), and a cytoplasmic C-terminus. S1-S4 are primary voltage sensor for channel opening and S5 and S6 are domains for ion pore forming

WES was performed on the proband as described previously (Fig. 2A: II-6). A novel heterozygous frameshift variant (NM_000238.4:c.3257_3258insG) was identified in exon 14 of the *KCNH2* gene, potentially explaining her prolonged QT interval. This variant encodes the α-subunit of the rapid delayed rectifier potassium channel (hERG), which is crucial for cardiac repolarization. The variant is predicted to cause a truncated protein due to the introduction of a premature stop codon at position 1119 (Gly1087Trpfs*32), potentially leading to a loss of function of the hERG channel. It causes a shift in the reading frame starting at codon 1087, changing the glycine amino acid to a tryptophan.

The p.Gly1087Trpfs*32 variant was absent from population databases (gnomAD, 1000 Genomes, and ExAC) and was not reported in disease-specific databases (ClinVar and HGMD). Based on the American College of Medical Genetics and Genomics (ACMG) guidelines, the p.Gly1087Trpfs32 variant was classified as likely pathogenic based on the following criteria: PP1, PP4, PP5, PM2, and PM4 [11].

Sanger sequencing confirmed the presence of the p.Gly1087Trpfs*32 variant in the proband (Fig. 2A: II-6) and its absence in the unaffected father (Fig. 2A: I-3). The deceased daughter (Fig. 2A: III-1) was not available for genetic testing. No other potentially pathogenic variants were identified in LQTS-associated genes in the proband.

## Discussion

In the present study, we identified a novel frameshift variant (p.Gly1087Trpfs*32) in C-terminal region of the *KCNH2* gene in a proband with LQTS and a family history of sudden cardiac death. The variant was classified as likely pathogenic based on ACMG guidelines and is predicted to result in a truncated protein due to the introduction of a premature stop codon.

The p.Gly1087Trpfs*32 variant, identified in this study, has not been previously reported in the scientific literature or in public databases such as 1000G projects, ClinVar, and GnomAD. According to Human Gene Mutation Database (HGMD; https://www.hgmd.cf.ac.uk/ac), as of January 2024, 959 mutations in the *KCNH2* gene have been identified, with 38 of these mutations associated with LQT2 syndrome. Approximately two-thirds of the reported *KCNH2* mutations are missense mutations, wherein a single change in the nucleotide sequence results in a defective amino acid causing loss of function of the Kv11.1ion channel.

The α-subunits of voltage-gated potassium channels, including Kv11.1, are membrane proteins composed of 4 homologous domains, each containing 6 α-helical transmembrane segments (S1 to S6) [12, 13]. *KCNH2* protein, also known as hERG or Kv11.1, consist of cytoplasmic N-terminus (NH2-terminus) and C-terminus (COOH-terminus) domains that harbor several some regulatory sites [14]. Although mutations in both the N- or C-terminal regions of the *KCNH2* potassium channel have been reported, most of the identified mutations in the *KCNH2* potassium channel gene encode amino acid changes located in the pore region [15, 16]. The Gly1087Trpfs*32 variant identified in our study is located in the C-terminal region of the KCNH2 protein. This region contains several important regulatory domains, including the cyclic nucleotide-binding domain (CNBD), an endoplasmic reticulum (ER) retention signal, and a conserved coiled-coil domain. These domains play crucial roles in various biophysical processes, such as modulation of I_Kr_ inactivation, protein trafficking, and subunit oligomerization (Fig. 2C) [14, 17].

Several studies have reported several disease-causing variants in the C-terminal region which can lead to altered channel kinetics, reduced surface expression, and impaired protein stability, highlighting the importance of this region in the pathogenesis of LQT2. For instance, in a study by Zio et al., a KCNH2 channel C-terminal variant, G1006fs/49, was identified in members of an Italian family with LQT2 [14]. This study revealed several important aspects of how C-terminal variants in KCNH2 can affect the channel’s function. First, the authors demonstrated that the G1006fs/49 variant protein was present in the plasma membrane, even when co-expressed with wild-type KCNH2. Second, they showed that the G1006fs/49 variant exerted a dominant negative effect on wild-type KCNH2, altering the biophysical properties of the heterotetrameric channel. Finally, they found that a specific KCNH2 activator could partially restore the activation kinetics of the G1006fs/49-containing heterotetrameric channels. Kupershmidt et al. cloned HERGUSO, a C-terminal splice variant, and demonstrated that a specific 104-amino acid domain in the C-terminus is critical for channel function [18]. Kupershmidt et al. in another study, identified an endoplasmic reticulum (ER) retention signal (RGR) at amino acids 1005–1007, which, when exposed by mutations truncating the HERG C-terminus, causes ER retention and reduced trafficking of the channel to the cell surface [19]. Nakajima et al. characterized a missense mutation (p.Arg534Cys) in the S4 region, suggesting its role as a voltage sensor and the impact of the mutation on channel gating properties [20]. Sasano et al. studied a novel C-terminus frameshift mutation (p.Asp1122Alafs*147) that generated additional 147 amino acids, resulting in reduced current density, accelerated inactivation, and a negative shift in steady-state inactivation [21]. Zhou et al. investigated various LQT2 mutations, revealing that the loss of HERG channel function can be caused by multiple mechanisms, including abnormal channel processing, generation of non-functional channels, and altered channel gating [22]. Several other hERG variants including R1014 × [23], P872fsX5 [24], G965X, R1014PfsX39, and V1038AfsX21 [25] have been identified in the C-terminus, result in protein truncation.

These studies are consistent with our findings in terms of the location of the variant. Similar to other reported c-terminal variants, it seems our identified variant also causes truncated protein followed by loss of protein function. Therefore, the Gly1087Trpfs*32variant can be one of the diagnostic markers for LQT2 disease and performing genetic tests can provide valuable information for early diagnosis in the LQTS patients and prognosis in relatives and treatment.

While our study provides strong evidence for the association of the novel p.Gly1087Trpfs*32 variant with LQT2 in the affected family, we acknowledge that functional characterization is necessary to definitively establish its pathogenicity and elucidate the underlying mechanisms.

## Conclusions

Through WES, we identified a novel heterozygous *KCNH2* frameshift mutation likely underlying LQTS in a 37-year-old Iranian woman. This premature stop-codon variant was classified as likely pathogenic by ACMG criteria. Located in the critical C-terminal region, this truncation likely disturbs regulatory interactions essential for proper IKr channel conduction. This report expands the mutational spectrum of LQT2 and exemplifies the utility of genomic sequencing in elucidating arrhythmia-associated genetic defects to enable early diagnosis and personalized management of cardiac channelopathies.

## Data Availability

The datasets generated and/or analyzed during the current study are available in the ClinVar repository [https://www.ncbi.nlm.nih.gov/clinvar/variation/2687478/]. The accession number of the variant in ClinVar is as follows: KCNH2 (NM_000238.4): c.3257_3258insG (Gly1087Trpfs*32): VCV002687478.1.
